# Perioperative chemotherapy (FLOT 4 + 4) versus total neoadjuvant therapy (TNT; FLOT ×8) for resectable gastric cancer: A systematic review and exploratory meta-analysis

**DOI:** 10.1016/j.sopen.2026.02.001

**Published:** 2026-02-26

**Authors:** Rafael Novaes Jardim, Vitório Augusto Alexandre Alves, Alessandro D. Mazzotta, Jayant Kumar, Omar Llaguna, Mohamed Ali Chaouch, Elio Pietro Perrone, Eulália Dalba Elias da Silva, Laura Correia Jacinto, Moacyr Jesus Barreto de Melo Rego, Nagy Habib, Adriano Carneiro da Costa

**Affiliations:** a Department of Surgery, Hospital of Clinics, Federal University of Pernambuco, Recife, PE, Brazil; b Department of General and Specialist Surgery and Anesthesiology, Sapienza University of Rome, Rome, Italy; c Memorial Healthcare, Hollywood, FL, USA; d Department of Visceral and Digestive Surgery, University of Monastir, Monastir, Tunisia; e Department of Translational and Precision Medicine, Sapienza University of Rome, Rome, Italy; f Therapeutic Innovation Research Center Suely Galdino, Federal University of Pernambuco, Recife, PE, Brazil; g Department of Surgery and Cancer, Hammersmith Hospital, Imperial College London, London, UK

**Keywords:** Gastric cancer, FLOT, Treatment completion, Pathological complete response, Survival

## Abstract

**Introduction:**

Gastric and gastroesophageal junction (GEJ) adenocarcinoma is associated with high recurrence rates despite curative-intent surgery. Standard perioperative chemotherapy (PC) using FLOT (4 preoperative + 4 postoperative cycles) improves survival but is frequently not completed because of postoperative morbidity. Total Neoadjuvant Therapy (TNT), which delivers all eight FLOT cycles preoperatively (FLOT ×8), has been proposed to improve treatment completion. This systematic review and meta-analysis compared TNT with standard perioperative FLOT (FLOT 4 + 4) in resectable gastric cancer.

**Methods:**

A systematic search following PRISMA 2020 guidelines was conducted in PubMed, Scopus, EMBASE, ScienceDirect, Cochrane Library, LILACS, and SpringerLink through September 2025. Comparative studies evaluating TNT versus PC in adults with resectable gastric or GEJ adenocarcinoma were included. Primary outcomes were chemotherapy completion and pathological complete response (pCR). Secondary outcomes included R0 resection, postoperative morbidity, treatment-related toxicity, overall survival (OS), and disease-free survival (DFS).

**Results:**

Three retrospective comparative studies including 425 patients (136 TNT, 289 PC) were analyzed. TNT was associated with higher completion of planned chemotherapy (OR 4.55; 95% CI 1.13–18.27; p < 0.01) without an increase in major postoperative morbidity (OR 0.96; 95% CI 0.55–1.69; p = 0.50). No significant differences were observed in pCR (OR 1.54; 95% CI 0.65–3.63) or R0 resection rates. Survival outcomes were heterogeneous and could not be reliably pooled.

**Conclusion:**

TNT was associated with improved chemotherapy completion without increased perioperative morbidity. However, the current evidence base is insufficient to support conclusions regarding oncologic efficacy or survival benefit.

## Highlights

• Total neoadjuvant FLOT (×8) increases completion of planned chemotherapy.
• Postoperative morbidity is similar to the perioperative FLOT (4 + 4) approach.
• No significant difference in pathological complete response was observed.
• Current evidence is limited to small retrospective studies with low certainty.
• Ongoing randomized trials will clarify the oncologic impact of TNT.

## Introduction

Gastric and gastroesophageal junction (GEJ) adenocarcinoma remains a major cause of cancer-related mortality worldwide [1], [2]. Despite advances in surgical techniques, locoregional treatment alone is frequently insufficient, as disease recurrence is commonly driven by occult micrometastatic spread [3]. These limitations have supported the adoption of multimodal treatment strategies.

The MAGIC trial established perioperative chemotherapy (PC) as part of curative-intent treatment [4], a concept further refined by the FLOT4-AIO trial. The FLOT regimen—fluorouracil, leucovorin, oxaliplatin, and docetaxel—administered as four preoperative and four postoperative cycles (FLOT 4 + 4), demonstrated a significant improvement in overall survival compared with previous standards [5], [6]. However, postoperative morbidity and delayed recovery frequently limit completion of adjuvant chemotherapy, with less than half of patients completing all eight planned cycles in the original trial [5], [7].

Total Neoadjuvant Therapy (TNT) has been proposed as an alternative strategy. By delivering all planned FLOT cycles before surgery (FLOT ×8), TNT aims to improve chemotherapy completion and reduce treatment attrition related to postoperative recovery [8]. Similar treatment sequencing strategies have been evaluated in rectal cancer, where preoperative intensification has been associated with improved treatment delivery [9], [10], [11], [12].

This systematic review and meta-analysis evaluates the safety, treatment completion, and clinical outcomes of TNT (FLOT ×8) compared with standard perioperative chemotherapy (FLOT 4 + 4) in patients with resectable gastric cancer.

## Methods

### Study design and registration

This systematic review and meta-analysis was conducted in accordance with the PRISMA 2020 guidelines [13] and prospectively registered in PROSPERO (CRD420251180402).

### Search strategy

A comprehensive electronic search was performed in PubMed, Scopus, EMBASE, ScienceDirect, Cochrane Library, LILACS, and SpringerLink from database inception through September 2025, without restrictions on language or publication date. The search strategy combined free-text terms and MeSH headings as follows: (“gastric cancer” OR “stomach neoplasms”) AND (“neoadjuvant chemotherapy” OR “total neoadjuvant”) AND (“perioperative chemotherapy” OR “perioperative”) AND (FLOT OR TNT). Duplicate records were removed using Rayyan software.

### Eligibility criteria

Eligibility was defined using the PICO framework. Included studies enrolled adult patients (≥18 years) with histologically confirmed, resectable, non-metastatic gastric or gastroesophageal junction adenocarcinoma. TNT was defined as delivery of all planned preoperative FLOT-based cycles (typically eight), and the comparator was standard perioperative FLOT (4 preoperative + 4 postoperative cycles). Primary outcomes were chemotherapy completion and pathological complete response. Secondary outcomes included R0 resection, severe postoperative morbidity (Clavien–Dindo ≥ III), treatment-related toxicity, overall survival, and disease-free survival. Both randomized and comparative observational studies were eligible.

### Study selection and data extraction

Two reviewers independently screened titles, abstracts, and full-text articles. Disagreements were resolved through consensus. Data extraction was performed independently using a standardized form capturing study design, patient demographics, tumor characteristics, treatment regimens, and outcome data.

### Risk of Bias Assessment

Methodological quality was assessed using the Newcastle–Ottawa Scale for cohort studies. Studies were classified as high (7–9 stars), moderate (4–6 stars), or low quality (0–3 stars). Interobserver agreement was evaluated using Cohen's kappa statistic (Table 1). (See Table 2, Table 3, Table 4.)

**Table 1:** Newcastle-Ottawa Scale evaluation criteria for cohort studies.

Domain	Assessment item
**Selection** (Maximum 4 stars)	1. Representativeness of the exposed cohort
	2. Selection of the non-exposed cohort
	3. Ascertainment of exposure (e.g., secure records, structured interviews)
	4. Demonstration that the outcome of interest was not present at the start of the study
**Comparability** (Maximum 2 stars)	1. Comparability of cohorts on the basis of the design or analysis, controlling for important confounding factors (e.g., Gleason score, tumor stage)
**Outcome** (Maximum 3 stars)	1. Assessment of outcome (e.g., independent blind assessment, record linkage)
	2. Was follow-up long enough for outcomes to occur
	3. Adequacy of follow-up of cohorts (e.g., losses to follow-up unlikely to introduce bias)

**Table 2:** Main characteristics of the included studies.

Study	Buckarma et al. (2025) [17]	Rencuzogullari et al. (2024) [8]	Yang et al. (2023) [18]
Study Design	Retrospective Cohort	Retrospective Cohort with Propensity Score Matching (PSM)	Retrospective Cohort
Country	USA	Turkey	USA
Cohort Size	202 (71 TNT vs. 131 PC)	74 (37 TNT vs. 37 PC, after PSM)	149 (28 TNT vs. 121 PC)
Chemotherapy Regimens	Heterogeneous (FLOT/FOLFOX, etc.)	Homogeneous (FLOT only)	Heterogeneous (FLOT, ECF, etc.)
Completion Rate	TNT associated with more cycles (median 8 vs 6)	TNT with significantly higher completion rate (89.1% vs 67.6%)	No difference in completion of *all* planned cycles
Pathological Complete Response (pCR)	No difference (20% vs 22%)	Trend favoring TNT (18.9% vs 8.1%, not signif.)	Trend favoring TNT (14% vs 5.8%, not signif.)
Recurrence-Free Survival (RFS)	Better with ≥6 chemo cycles (favoring TNT strategy)	No difference	No difference (trend towards worse in TNT)
Overall Survival (OS)	Better with ≥6 chemo cycles (favoring TNT strategy)	No difference	No difference (trend towards worse in TNT)
Key Limitations	Selection bias, retrospective data	Relatively short follow-up, small N after PSM	Selection bias, small N in TNT

**Table 3:** Risk of Bias Assessment using the Newcastle-Ottawa Scale (NOS).

Study	Selection (max 4 stars)	Comparability (max 2 stars)	Outcome (max 3 stars)	Total score	Quality rating
Buckarma et al. (2025) [17]	****	*	***	8	High
Rencuzogullari et al. (2024) [8]	****	**	***	9	High
Yang et al. (2023) [18]	***		***	6	Moderate

**Table 4:** Baseline patient characteristics in included studies.

Study	Buckarma et al. (2025) [17]		Rencuzogullari et al. (2024) [8]		Yang et al. (2023) [18]	
**Chemotherapy Regimens**	**PC (N** **=** **131)**	**TNT (N** **=** **71)**	**PC (N** **=** **37)**	**TNT (N** **=** **37)**	**PC (N** **=** **121)**	**TNT (N** **=** **28)**
**Median Age, years (IQR/Range)**	63.0 (54.0, 71.0)	64.0 (56.0, 73.0)	62 [37–83]	64 [42–77]	63	64
**Gender (% Male)**	65.6%	70.4%	67.6%	67.6%	64%	54%
**ECOG Performance Status (% 0)**	66.4%	62.5%	Not reported	Not reported	58%	46%
**Primary Tumor Site**						
*Upper/Proximal*	Not reported	Not reported	24.3%	21.6%	29%	39%
*Middle*	Not reported	Not reported	(Part of distal)	(Part of distal)	36%	25%
*Lower/Distal*	Not reported	Not reported	75.7%	78.4%	36%	36%
**Clinical Stage (AJCC 8th Ed.****[19]****)**						
*Stage I*	6.1%	8.4%	5.4%	10.8%	Not reported	Not reported
*Stage II*	44.3%	46.5%	29.7%	43.2%	Not reported	Not reported
*Stage III*	49.6%	45.1%	64.9%	46.0%	Not reported	Not reported
**Clinical T Stage**						
*cT1/cT2*	Not reported	Not reported	Not reported	Not reported	4.8%	10.6%
*cT3*	Not reported	Not reported	Not reported	Not reported	58%	58%
*cT4*	Not reported	Not reported	Not reported	Not reported	38%	32%
**Clinical N Stage (% cN+)**	Not reported	Not reported	Not reported	Not reported	56%	68%
**Lauren Histology****[20]**						
*Diffuse*	75.9%	43.5%	48.6%	35.1%	33%	36%
*Intestinal*	24.1%	56.5%	51.4%	64.9%	50%	41%
*Mixed*	Not reported	Not reported	Not reported	Not reported	17%	23%
**WHO Histology (% Poorly Diff.)**	Not reported	Not reported	Not reported	Not reported	69%	75%
**Preoperative Chemotherapy**						
*FLOT/Docetaxel-based*	49.6%	87.3%	100%	100%	31%	79%
*5FU/Platinum*	(Part of modern)	(Part of modern)	0%	0%	40%	14%
*Epirubicin-based (“Magic”)*	47.3%	9.9%	0%	0%	29%	7.1%

Note: Percentages may not sum to 100 due to rounding, mixed categories, or missing data in the source studies. Clinical stage data for Buckarma et al. and Rencuzogullari et al. were aggregated from detailed staging (e.g., IIA, IIB) for clarity. ECOG = Eastern Cooperative Oncology Group; PSM = Propensity Score Matched.

### Statistical analysis

Meta-analyses were conducted in R (meta package) using random-effects Mantel–Haenszel models. Odds ratios with 95% confidence intervals were calculated for binary outcomes. Heterogeneity was assessed using the I^2^ statistic. Interobserver agreement demonstrated excellent reliability (κ = 0.864).

## Results

### Study selection

**Fig. 1:**
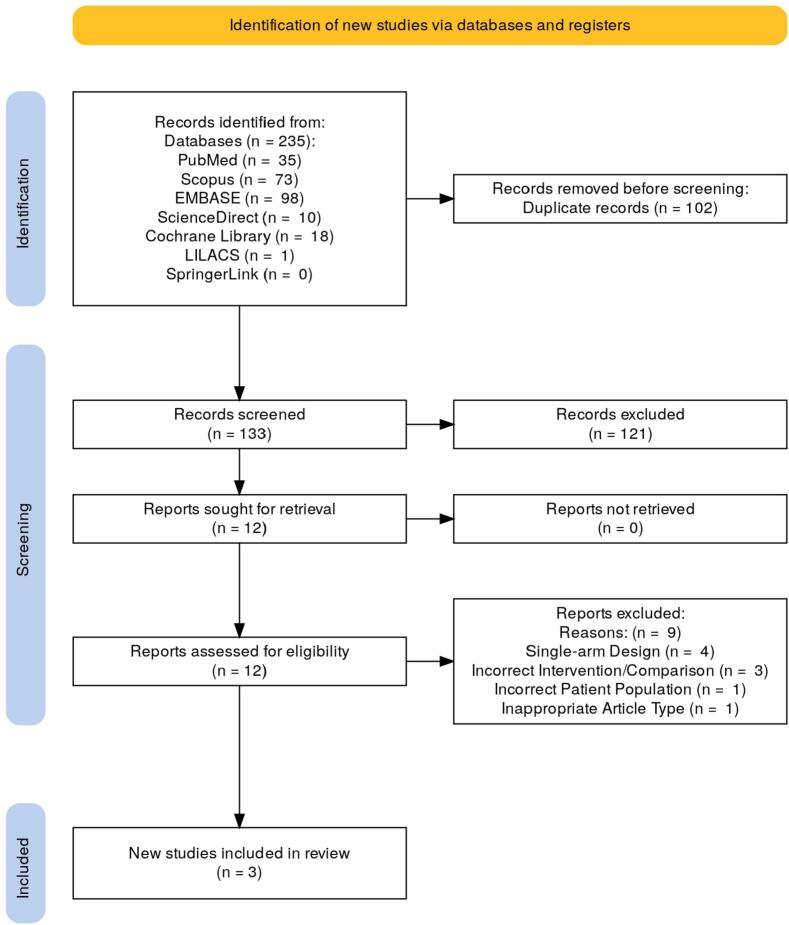
Process of searching and selecting studies for the systematic review, following PRISMA guidelines.

The literature search identified 235 records. After duplicate removal and screening, 12 full-text articles were assessed, of which 9 were excluded based on predefined criteria. Three retrospective comparative studies were included in the qualitative and quantitative synthesis. No randomized controlled trials were identified (Fig. 1).

### Study characteristics and risk of bias

**Fig. 2:**
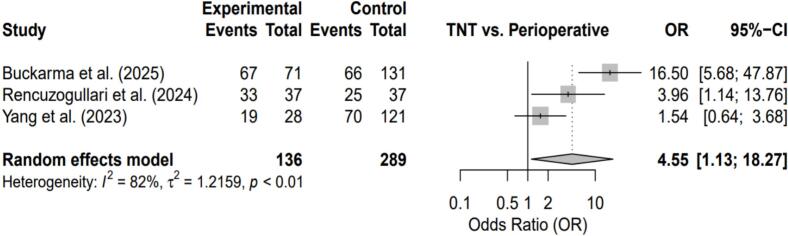
Forest plot comparing Total Neoadjuvant Therapy (TNT) versus Perioperative Chemotherapy (PC) for treatment completion rate.

**Fig. 3:**
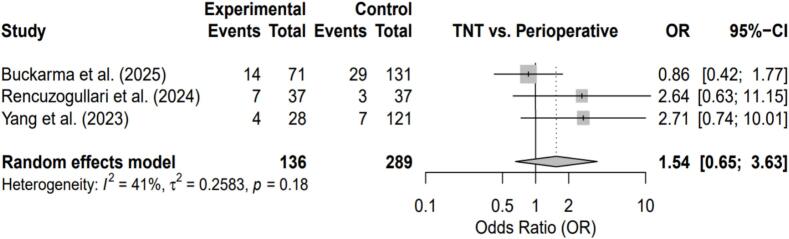
Forest plot comparing Total Neoadjuvant Therapy (TNT) versus Perioperative Chemotherapy for pathological complete response (pCR) rate.

**Fig. 4:**
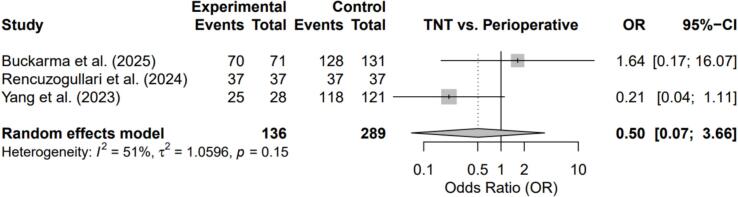
Forest plot comparing Total Neoadjuvant Therapy (TNT) vs. Perioperative Chemotherapy: R0 Resection Rate**.**

**Fig. 5:**
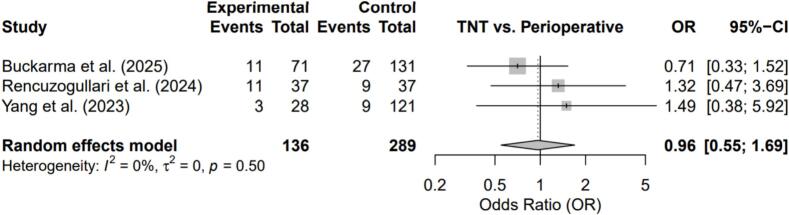
Forest plot comparing TNT versus PC for severe postoperative morbidity (Clavien–Dindo grade ≥ III).

The three included studies, published between 2023 and 2025, comprised a total of 425 patients (136 TNT and 289 perioperative chemotherapy) from Turkey and the United States. One study applied propensity score matching to improve baseline comparability, while two used unmatched retrospective designs. Two studies were rated as high quality and one as moderate quality, with selection bias identified as a major concern in the latter (Fig. 2, Fig. 3, Fig. 4, Fig. 5).

### Patient and clinical characteristics

Across studies, median patient age ranged from 62 to 64 years, with a predominance of male patients. Most participants presented with locally advanced disease (stage II–III). Notable heterogeneity was observed in histopathological subtype distribution and chemotherapy regimens, particularly in unmatched cohorts, where perioperative groups frequently received non-FLOT regimens.

### Meta-analysis outcomes

#### Treatment completion

Patients treated with TNT were significantly more likely to complete all eight planned chemotherapy cycles compared with those receiving perioperative chemotherapy (OR = 4.55; 95% CI, 1.13–18.27; p < 0.01). Substantial heterogeneity was observed (I^2^ = 82%).

#### Pathological complete response

No statistically significant difference in pCR rates was observed between TNT and perioperative chemotherapy (OR = 1.54; 95% CI, 0.65–3.63; p = 0.18), with moderate heterogeneity (I^2^ = 41%).

### Surgical outcomes

There were no significant differences in R0 resection rates (OR = 0.50; 95% CI, 0.07–3.66; p = 0.15) or major postoperative morbidity (OR = 0.96; 95% CI, 0.55–1.69; p = 0.50). Heterogeneity was moderate for R0 resection and absent for postoperative complications (I^2^ = 0%).

### Survival outcomes

A quantitative meta-analysis of OS and DFS was not performed due to heterogeneity in reporting methods, variation in follow-up duration, and high risk of bias. Survival outcomes were therefore not considered primary endpoints of this synthesis.

## Discussion

### Principal findings

This systematic review and exploratory meta-analysis synthesized the available comparative evidence from three retrospective cohorts evaluating Total Neoadjuvant Therapy (TNT) using FLOT in resectable gastric cancer. Two consistent findings were identified. First, TNT was associated with a significantly higher likelihood of completing all planned chemotherapy cycles compared with perioperative chemotherapy (PC) [8], [17]. This finding supports the rationale that delivering systemic therapy entirely in the preoperative phase improves treatment adherence by avoiding postoperative delays, treatment interruptions, and attrition related to surgical recovery. Second, TNT was not associated with an increased incidence of major postoperative morbidity (Clavien–Dindo grade ≥ III) [8], [17], [18], indicating that intensified preoperative chemotherapy can be delivered without compromising short-term surgical safety.

Despite these improvements in treatment delivery, current evidence does not demonstrate a definitive oncologic advantage for TNT. No consistent differences were observed in pathological complete response or survival-related outcomes across the included studies [8], [17], [18]. Interpretation of these endpoints is limited by the absence of randomized controlled trials, heterogeneity in chemotherapy regimens and comparator arms, variability in follow-up duration, and the retrospective nature of the available data.

Given these limitations, the quantitative synthesis should be interpreted with caution. The small number of included studies and modest pooled sample size resulted in wide confidence intervals and reduced statistical precision. Under these conditions, pooled estimates remain exploratory and may be sensitive to the inclusion of additional studies. Consequently, these results should be considered hypothesis-generating, and greater interpretative weight should be placed on consistent qualitative patterns rather than on numerical point estimates alone.

### Treatment delivery and oncologic uncertainty

The most consistent finding across the included studies was the improvement in chemotherapy completion achieved with TNT, directly addressing a major limitation of PC related to postoperative treatment attrition [8]. In conventional perioperative strategies, postoperative complications, delayed recovery, and deterioration in performance status frequently limit the administration of adjuvant chemotherapy. Shifting all systemic treatment to the preoperative phase appears to mitigate this limitation by improving treatment adherence and dose intensity.

However, this improvement in treatment delivery was not accompanied by clear improvements in downstream oncologic endpoints. This discrepancy highlights that enhanced chemotherapy completion does not necessarily translate into measurable gains in pathological response or survival outcomes within the constraints of the current evidence base. Selection bias, immortal time bias, and heterogeneity in chemotherapy regimens further complicate interpretation and may obscure true treatment effects.

From an evidence-based perspective, TNT can therefore be considered feasible and safe, but its therapeutic effectiveness remains unproven. Establishing a causal relationship between treatment sequencing and oncologic outcomes will require adequately powered randomized trials with standardized FLOT-based protocols, consistent outcome definitions, and sufficient follow-up duration.

### Implications for clinical practice

The findings of this review suggest that TNT represents a feasible strategy to improve chemotherapy completion while maintaining acceptable perioperative safety in patients with resectable gastric cancer [8], [18]. By addressing a major limitation of PC, TNT may offer practical advantages in treatment delivery. However, in the absence of high-quality prospective evidence demonstrating oncologic benefit, TNT should not be adopted as routine clinical practice.

At present, TNT should be regarded as an investigational strategy and its use should remain restricted to clinical trial settings or carefully selected institutional protocols with appropriate multidisciplinary oversight and patient counseling.

### Ongoing studies and future research

A randomized pilot trial (NCT05567835) designed to compare TNT (FLOT ×8) with standard perioperative FLOT (FLOT 4 + 4) was terminated before completion, leaving a gap in prospective comparative data. Consequently, ongoing randomized trials, particularly OCTASUR (NCT06028737) [21], are expected to provide critical evidence regarding feasibility, safety, and long-term oncologic outcomes in patients with resectable gastric and gastroesophageal junction cancer.

Future studies should prioritize standardized chemotherapy protocols, intention-to-treat analyses, robust reporting of survival endpoints, and assessment of patient-centered outcomes, including treatment tolerance, postoperative recovery, and quality of life.

## Limitations

The primary limitation of this review is the absence of randomized controlled trials, with all available evidence derived from small, retrospective, and methodologically heterogeneous studies. Although a meta-analysis was performed due to the scarcity of comparative data, the limited number of studies and modest pooled sample size resulted in wide confidence intervals and reduced statistical precision, rendering the pooled estimates exploratory and statistically fragile. Under these conditions, survival-related outcomes in particular are vulnerable to imprecision and instability.

Selection bias further limits interpretability, as TNT cohorts were frequently enriched with patients demonstrating favorable early treatment tolerance or response. This introduces the potential for immortal time bias, since patients must survive and remain fit throughout the prolonged neoadjuvant period in order to be classified within the TNT group. These methodological artifacts may artificially inflate apparent pathological response and survival estimates independent of true treatment effect.

Additional confounding arises from heterogeneity in perioperative chemotherapy regimens, with a substantial proportion of comparator patients receiving non-FLOT protocols. This compromises internal validity by introducing regimen-related efficacy differences unrelated to treatment sequencing. Moreover, the lack of consistent intention-to-treat analyses and insufficient disaggregated data prevented sensitivity analyses restricted to uniformly treated FLOT cohorts, further limiting the ability to isolate the independent impact of treatment timing.

Collectively, these limitations indicate that the current evidence base is underpowered to reliably detect modest but clinically meaningful oncologic benefit and underscore the necessity for adequately powered prospective randomized trials with standardized treatment protocols and long-term follow-up.

## Conclusion

Total Neoadjuvant Therapy (TNT) using the FLOT regimen has been associated with higher chemotherapy completion rates compared with the standard perioperative approach, without an apparent increase in surgical morbidity. However, this observation reflects improved treatment delivery rather than evidence of oncologic effectiveness.

Because the current evidence is limited to small retrospective studies with significant methodological constraints and potential bias, no reliable conclusions can be drawn regarding pathological response, disease control, or long-term survival outcomes. As a result, TNT cannot be considered oncologically equivalent or superior to conventional perioperative strategies based on the available data.

The outcomes of ongoing randomized controlled trials, particularly the OCTASUR trial (NCT06028737), are essential to clarify whether higher treatment completion rates translate into clinically meaningful oncologic benefit. Until robust prospective evidence becomes available, TNT should be regarded as an investigational approach and its routine use should remain restricted to controlled clinical trial settings.

## CRediT authorship contribution statement

**Rafael Novaes Jardim:** Methodology, Investigation. **Vitório Augusto Alexandre Alves:** Visualization, Validation, Methodology. **Alessandro D. Mazzotta:** Writing – review & editing, Writing – original draft, Methodology, Investigation. **Jayant Kumar:** Validation, Investigation, Formal analysis. **Omar Llaguna:** Visualization. **Mohamed Ali Chaouch:** Writing – review & editing, Visualization, Data curation. **Elio Pietro Perrone:** Visualization. **Eulália Dalba Elias da Silva:** Writing – review & editing, Writing – original draft, Visualization. **Laura Correia Jacinto:** Writing – original draft, Visualization, Methodology. **Moacyr Jesus Barreto de Melo Rego:** Writing – review & editing, Visualization. **Nagy Habib:** Writing – review & editing, Visualization, Validation. **Adriano Carneiro da Costa:** Writing – review & editing, Writing – original draft, Methodology, Data curation.

## Declaration of Generative AI and AI-assisted technologies in the writing process

During the preparation of this manuscript, the authors used ChatGPT (OpenAI) exclusively for language refinement. All scientific content, data interpretation, and conclusions were generated entirely by the authors.

## Funding

This research received no specific grant from any funding agency in the public, commercial, or not-for-profit sectors.

## Declaration of competing interest

The authors declare no conflicts of interest.

## Data Availability

All data analyzed in this systematic review are derived from previously published studies available in public databases. No new primary data were generated.
